# The Tumorigenicity of Mouse Embryonic Stem Cells and *In Vitro* Differentiated Neuronal Cells Is Controlled by the Recipients' Immune Response

**DOI:** 10.1371/journal.pone.0002622

**Published:** 2008-07-09

**Authors:** Ralf Dressel, Jan Schindehütte, Tanja Kuhlmann, Leslie Elsner, Peter Novota, Paul Christian Baier, Arne Schillert, Heike Bickeböller, Thomas Herrmann, Claudia Trenkwalder, Walter Paulus, Ahmed Mansouri

**Affiliations:** 1 Department of Cellular and Molecular Immunology, University of Göttingen, Göttingen, Germany; 2 Department of Clinical Neurophysiology, University of Göttingen, Göttingen, Germany; 3 Department of Neuropathology, University of Göttingen, Göttingen, Germany; 4 Institute of Neuropathology, University Hospital Münster, Münster, Germany; 5 Department of Genetic Epidemiology, University of Göttingen, Göttingen, Germany; 6 Institute of Medical Biometry and Statistics, University of Lübeck, Lübeck, Germany; 7 Institute of Virology and Immunobiology, University of Würzburg, Würzburg, Germany; 8 Paracelsus-Elena Klinik, Kassel, Germany; 9 Department of Molecular Cell Biology, Max-Planck-Institute for Biophysical Chemistry, Göttingen, Germany; Centre de Regulacio Genomica, Spain

## Abstract

Embryonic stem (ES) cells have the potential to differentiate into all cell types and are considered as a valuable source of cells for transplantation therapies. A critical issue, however, is the risk of teratoma formation after transplantation. The effect of the immune response on the tumorigenicity of transplanted cells is poorly understood. We have systematically compared the tumorigenicity of mouse ES cells and *in vitro* differentiated neuronal cells in various recipients. Subcutaneous injection of 1×10^6^ ES or differentiated cells into syngeneic or allogeneic immunodeficient mice resulted in teratomas in about 95% of the recipients. Both cell types did not give rise to tumors in immunocompetent allogeneic mice or xenogeneic rats. However, in 61% of cyclosporine A-treated rats teratomas developed after injection of differentiated cells. Undifferentiated ES cells did not give rise to tumors in these rats. ES cells turned out to be highly susceptible to killing by rat natural killer (NK) cells due to the expression of ligands of the activating NK receptor NKG2D on ES cells. These ligands were down-regulated on differentiated cells. The activity of NK cells which is not suppressed by cyclosporine A might contribute to the prevention of teratomas after injection of ES cells but not after inoculation of differentiated cells. These findings clearly point to the importance of the immune response in this process. Interestingly, the differentiated cells must contain a tumorigenic cell population that is not present among ES cells and which might be resistant to NK cell-mediated killing.

## Introduction

Embryonic stem (ES) cells are a potential source of cells and tissues for transplantation in regenerative medicine. However, one of the critical issues is the risk of teratoma formation after transplantation of ES cells. It has been reported, e. g., that undifferentiated mouse ES cells can develop into functional dopaminergic neurons after intrastriatal transplantation in a rat model of Parkinson's disease but teratomas occurred in about 20% of the recipients which had been treated with cyclosporine A (CsA) for immunosuppression [1]. Transplantation of dopaminergic neurons differentiated *in vitro* from ES cells improved amphetamine-induced rotational behavior in the unilaterally 6-hydroxy-dopamine (6-OHDA)-lesioned rat model for Parkinson's disease [2]. These rats which were continuously treated with CsA did not develop teratomas [2]. Functional improvements without the development of teratomas have been observed after transplantation of neuronal cells differentiated from ES cells on PA6 feeder cells into the striata of 6-OHDA-lesioned rats which had not received any immunosuppressive treatment [3]. Despite the behavioral changes of the transplanted animals, the grafted cells remained in compact deposits surrounded by glia cells without functional integration into the host tissue [3], which is postulated for an optimal long-term survival of grafts. When these *in vitro* differentiated neuronal cells were transplanted into CsA-treated recipients, tyrosine hydroxylase (TH)-positive neurites were present in the grafts suggesting a better integration of transplanted cells, however, now teratomas occurred in 2 of 15 animals [4]. In all these experiments a xenotransplantation was performed because rat ES cells are not readily available whereas the rat model allows for a reliable functional evaluation of grafts. The results might suggest that immunosuppression is required for functional integration of grafted cells but is associated with the risk of teratoma formation. Systematic comparative studies which address these questions are lacking. In one study a higher prevalence of teratomas was observed after intracerebral transplantation of ES cells in CsA-treated mice than in rats suggesting that the tumorigenesis of ES cells partially depends on the host [5]. Teratomas have been found also after injection of ES or *in vitro* differentiated cells into various other tissues including, e.g., liver [6] and myocardium [7]–[9].

It has been proposed that teratoma formation can be prevented by *in vitro* pre-differentiation of ES cells [2] although conflicting results have been reported as well [4], [5]. In accordance with this hypothesis transplantation of ES cells into immunosuppressed allogeneic mice frequently leads to teratomas but *in vitro* pre-differentiation can reduce the tumorigenicity of the grafts [10]. Sorting of cells expressing the neural precursor marker Sox1 before transplantation has been shown to further reduce the risk of teratoma formation [10], [11]. Furthermore, it has been reported that neuronal precursors can be enriched by inducing apoptosis in pluripotent stem cells using ceramide analogues so that teratoma formation is avoided [12]. These results are compatible with the common hypothesis that only undifferentiated stem cells can give rise to teratomas and that teratoma formation after injection of differentiated cells is caused by contamination of the grafts with undifferentiated cells.

In general, the effect of the immune response on the tumorigenicity of transplanted undifferentiated ES cells and *in vitro* differentiated cells is important but still poorly understood. Therefore, we systematically compared the tumorigenicity of mouse ES cells and *in vitro* differentiated neuronal cells after subcutaneous injection in immunocompetent and immunosuppressed syngeneic, allogeneic, and xenogeneic hosts.

## Results

### Tumorigenicity of ES cells and differentiated cells in syngeneic but not in allogeneic or xenogeneic hosts

We analyzed the tumorigenicity of ES cells (MPI-II) and ES cell-derived neuronal cells which were differentiated *in vitro* for 14 days in syngeneic, allogeneic, and xenogeneic hosts. Since we expected that ES cells would give rise to teratomas in syngeneic 129Sv mice, we determined the number of ES cells that were necessary to achieve tumor growth after subcutaneous injection in the majority of mice. Within 100 days tumors were observed in all mice which received at least 1×10^6^ ES cells (Table 1). Therefore, the subsequent experiments were performed with a dose of 1×10^6^ cells.

**Table 1:** Tumor formation after subcutaneous inoculation of various numbers of ES cells or *in vitro* differentiated cells in 129Sv mice.

Cell number	ES cells	differentiated cells (d 14)
2×10^6^	100% (6/6)	100% (6/6)
1×10^6^	100% (6/6)	83% (5/6)
5×10^5^	33% (2/6)	17% (1/6)
1×10^5^	17% (1/6)	0% (0/6)

The indicated number of ES cells and cells differentiated *in vitro* for 14 days were injected subcutaneously into the flank of 129Sv mice. The percentage and number of animals is indicated in which tumors were found during autopsy at the side of injection before day 100 after injection.

**Figure 1:**
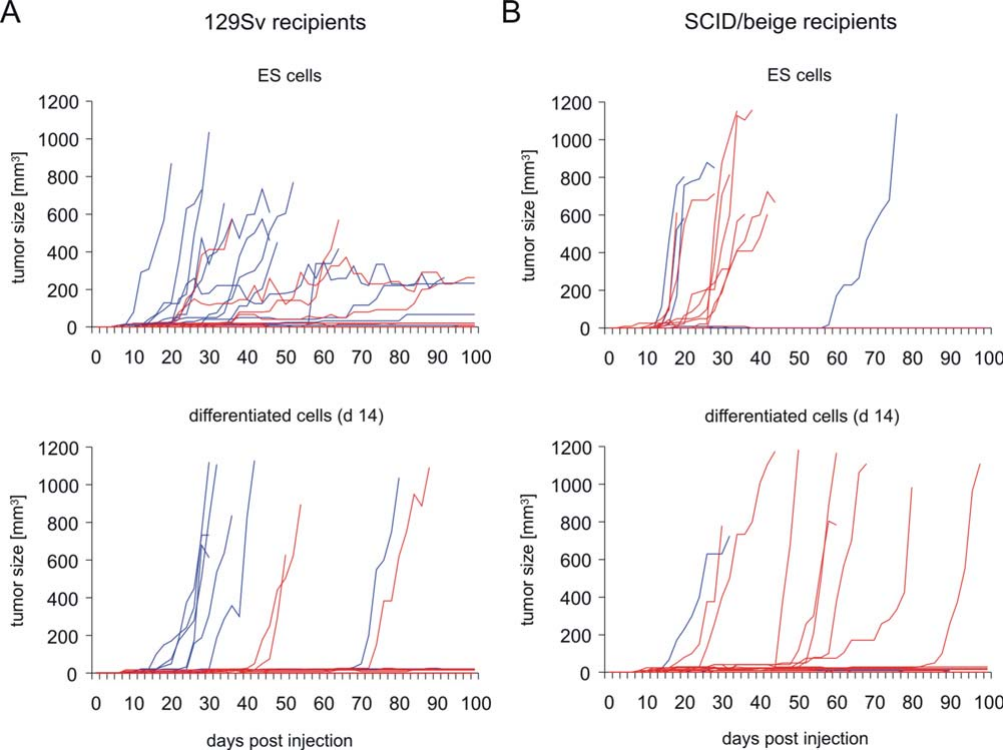
Tumor growth in syngeneic 129Sv and immunodeficient SCID/beige mice after injection of ES cells or *in vitro* differentiated cells. (A) 1×10^6^ ES cells (MPI-II) or *in vitro* differentiated cells (day 14 of differentiation culture) were injected subcutaneously at day 0 into syngeneic 129Sv mice (for the numbers of animals see Table 2). The tumor size was recorded every second day until day 100 using linear calipers. The growth of tumors in individual mice is shown. Tumor growth in female hosts is indicated by red and in male hosts by blue lines. (B) 1×10^6^ ES cells or *in vitro* differentiated cells were injected subcutaneously at day 0 into immunodeficient SCID/beige mice which are deficient for T, B, and functional NK cells.

Syngeneic (129Sv), and allogeneic (C57BL/6 and C3H/HeN) mice as well as xenogeneic (LOU/c) rats were used as recipients. 129Sv and C57BL/6 mice share the major histocompatibility complex (MHC) haplotype H2^b^. These mice differ therefore only in minor histocompatibility antigens, but not in the major histocompatibility antigens, i.e., the classical MHC class I and class II molecules. C3H/HeN mice carry the MHC haplotype H2^k^ and differ in major and minor histocompatibility antigens from 129Sv mice. Injection of 1×10^6^ ES cells or differentiated cells into syngeneic hosts resulted in about 95% of the animals in palpable tumors at the site of injection within 100 days post injection (Table 2). In a few cases tumors that were palpable at least in 3 consecutive observations did not continue to grow and disappeared before day 100. Tumors grew early and rapidly in some animals, whereas small tumors remained stable for the whole observation time in others (Fig. 1). We did not observe a statistically significant difference in the growth of tumors after injection of ES and differentiated cells, although a trend (p = 0.0673) towards a reduced growth of differentiated cells was observable (Fig. 1). The growth of tumors was accelerated in male compared to female hosts (p<0.0001) after injection of the male ES cells (XY karyotype). We then determined the frequency of tumor growth after injection of various cell numbers of differentiated cells and observed a similar cell dose dependency as before for ES cells (Table 1).

**Table 2:** Tumor formation after subcutaneous inoculation of ES or *in vitro* differentiated cells into various hosts.

host	CsA	ES cells	differentiated cells (d 14)
syngeneic (129Sv, H2^b^)	−	96% (25/26)^1^	95% (21/22)^2^
allogeneic (C57BL/6, H2^b^)	−	0% (0/19)	0% (0/25)
allogeneic (C3H, H2^k^)	−	0% (0/13)	0% (0/21)
xenogeneic (LOU/c, RT1^u^)	−	0% (0/12)	0% (0/17)
allogeneic SCID/beige (C.B-17, H2^d^)	−	93% (13/14)^3^	94% (17/18)^4^
syngeneic (129Sv, H2^b^)	+	94% (15/16)^5^	83% (10/12)
allogeneic (C57BL/6, H2^b^)	+	0% (0/12)	8% (1/13)
allogeneic (C3H, H2^k^)	+	7% (1/15)	9% (1/11)
xenogeneic (LOU/c, RT1^u^)	+	0% (0/25)	61% (11/18)^6^

ES cells and cells differentiated *in vitro* for 14 days were injected subcutaneously into the flank of syngeneic or allogeneic mice or xenogeneic rats (1×10^6^ cells in PBS/animal). Some recipients received an immunosuppressive treatment with CsA (10 mg/kg/day). The percentage and number of animals in which tumors were found during autopsy or in which tumors were palpable (at least during 3 consecutive observations) at the side of injection before day 100 after injection is indicated.

1 in 2 mice (8%) a tumor regression was observed before day 100.

2 in 2 mice (9%) a tumor regression was observed before day 100.

3 in 1 mouse (7%) a tumor regression was observed before day 100.

4 in 2 mice (11%) a tumor regression was observed before day 100.

5 in 8 female mice (47%) a tumor regression was observed before day 100.

6 in 2 rats (11%) a tumor regression was observed before day 100.

**Figure 2:**
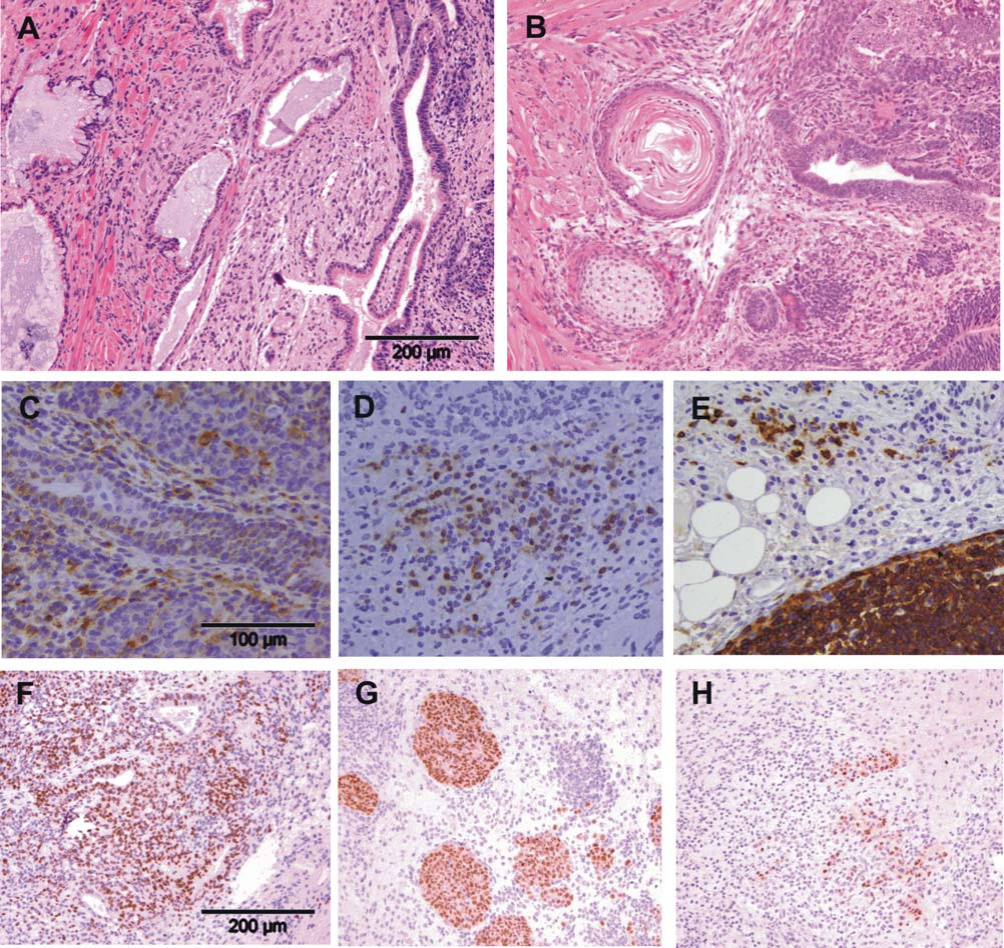
Histopathological analysis identifies tumors from ES and *in vitro* differentiated cells as teratomas. Tumors grown in 129Sv mice after injection of ES cells (A) or after injection of *in vitro* differentiated cells (B) were HE stained. The tumors are teratomas which show various differentiations. Teratomas from 129Sv mice were stained by immunohistochemistry for macrophages (C), T lymphocytes (D), B lymphocytes (E), and the proliferation marker Ki67 (F). Some teratomas grown in SCID/beige mice (G) and CsA-treated rats (H) expressed OCT3/4, a marker of pluripotent cells.

At the end of the experiments all animals underwent autopsy. No signs of regional or distant metastases were found. Histopathological evaluation identified all tumors as teratomas (Fig. 2A, B). Derivatives of the three germ layers were regularly observed during histopathological analysis of the tumors arising after injection of ES as well as *in vitro* differentiated cells. In agreement with the rapid growth of the tumors, the proliferation marker Ki67 was regularly detected by immunohistochemistry (Fig. 2F, Table 3). The pluripotency marker OCT3/4 (POU5F1) was not found to be expressed (Table 3). T and B lymphocytes as well as macrophages were present in most teratomas (Fig 2C–E, Table 3) but obviously not able to prevent tumor growth in syngeneic mice. In several teratomas B cells appeared even to form lymph follicle-like structures (Fig. 2E). The composition of teratomas with neuronal and glial cells was not different when tumors arising from ES cells and differentiated cells were compared (Table 4).

**Table 3:** Results of immunohistochemical stainings of teratomas grown in various hosts.

host	CsA	cells	OCT3/4	Ki67	T cells	B cells	macrophages
129Sv	−	ES	−	++	− to +++	+ to +++	+
129Sv	−	diff.	−	++	− to ++	− to +++	+
129Sv	+	ES	−	++	− to ++	− to +++	+
129Sv	+	diff.	−	++^1^	++ to +++	++ to +++	+
SCID/beige	−	ES	− to ++	++	n. t.^2^	n. t.	+
SCID/beige	−	diff.	− to ++	++^1^	n. t.	n. t.	+
LOU/c^3^	+	diff.	− to +	++^1^	+ to +++	++ to +++	++ to +++^1^

Teratomas were obtained after injection of ES cells or differentiated (diff.) cells into syngeneic 129Sv mice, immunodeficient SCID/beige mice or LOU/c rats. Some recipients received CsA (10 mg/kg/day). Histological sections were stained by immunohistochemistry for OCT3/4, Ki67, T cells (anti-mouse or anti-rat CD3), B cells (anti-mouse CD45R/B220 or anti-rat CD45R), and macrophages (anti-mouse Mac3 or anti-rat CD68). The presence of the markers is indicated semi quantitatively in four categories: − negative, + single positive cells, ++ groups of positive cells, +++ confluent groups of positive cells. Five representative teratomas per group of mice and 9 teratomas of LOU/c rats were analyzed.

1 One not rapidly growing tumor in each of these groups was negative for the marker.

2 not tested.

3 Teratomas grown in LOU/c rats were stained for infiltrating rat T and B cells as well as rat macrophages.

**Table 4:** Expression of neuronal and glial markers in teratomas grown in 129Sv and SCID/beige mice after injection of ES and differentiated cells.

host	cells	NeuN	GFAP
129Sv	ES	+ to +++	− to +++^1^
129Sv	diff.	− to ++^2^	− to +++^1^
SCID/beige	ES	− to +++^1^	+ to ++
SCID/beige	diff.	+to +++	++ to +++

Teratomas were obtained after injection of ES cells or differentiated (diff.) cells into syngeneic 129Sv mice and immunodeficient SCID/beige mice. Histological sections were stained by immunohistochemistry for the neuronal marker NeuN and the glial marker GFAP. The presence of the markers is indicated semi quantitatively in four categories: − negative, + single positive cells, ++ groups of positive cells, +++ confluent groups of positive cells. Five representative teratomas per group were analyzed.

1 2 of 5 teratomas were negative for this marker.

2 3 of 5 teratomas were negative for this marker.

Both cell types, ES and differentiated cells, did not give rise to tumors in xenogeneic LOU/c rats and in completely allogeneic C3H/HeN mice (Table 2). Differences in minor histocompatibility antigens were sufficient to prevent progressive tumor growth because C57BL/6 recipients did also not develop tumors (Table 2). Thus, the immune system appears to be able to prevent tumor growth after injection of ES and differentiated cells into allogeneic or xenogeneic hosts.

### Low frequency of undifferentiated cells in the differentiation cultures

**Figure 3:**
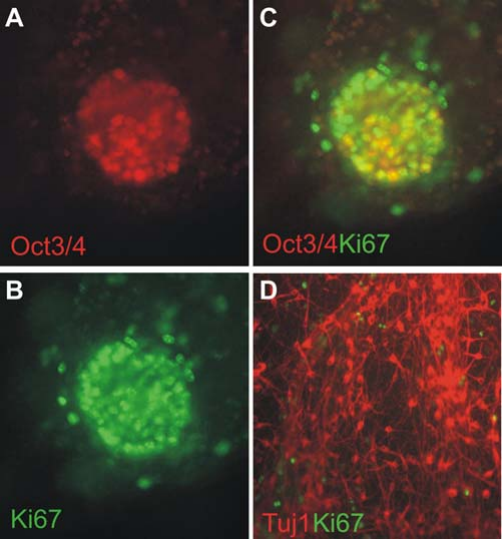
Some of the ES cell-derived neuronal colonies still contain at day 14 OCT3/4 and Ki67-positive cells. (A) After 14 days of differentiation culture few colonies (less than 5%), as exemplified here, still contain OCT3/4-positive cell clusters as detected by immunofluorescence staining using a specific mAb (magnification 40×). (B) The same colony was stained by an anti-Ki67 mAb to detect proliferating cells. (C) The merged staining indicates that Ki67-positive proliferating cells exist which are not OCT3/4-positive stem cells. (D) In parallel, cells of the neuronal differentiation culture were stained at day 14 by anti-Ki67 and anti-Tuj1 mAb. The merged staining (magnification 20×) indicates that the vast majority of the cells are Tuj1-positive neuronal cells. Few Ki67-positive proliferating cells are negative for the neuronal cell marker.

In syngeneic recipients both cell types led to tumors in similarly high frequency (∼95%), indicating the presence of tumorigenic cells also after differentiation culture. However, in accordance with our previous data [3] less than 5% of the colonies in the differentiation cultures at day 14 contained cells positive for OCT3/4 and Ki67 (Fig. 3A–C) which might represent still undifferentiated ES cells. Ki67-positive but OCT3/4-negative cells were also found in these rare colonies (Fig. 3C). Importantly, the vast majority of the cells in the differentiation cultures were III β-tubulin (Tuj1)-positive indicating a neuronal differentiation (Fig. 3D). The few proliferating Ki67-positive cells were negative for neuronal markers (Fig. 3D). In view of these characteristics of the *in vitro* differentiated cells, it was surprising that teratomas were equally frequent in syngeneic mice after injection of this heterogeneous but mainly neuronal cell population and a homogenous undifferentiated ES cell population.

### Tumorigenicity of ES cells and differentiated cells in allogeneic immunodeficient hosts

**Figure 4:**
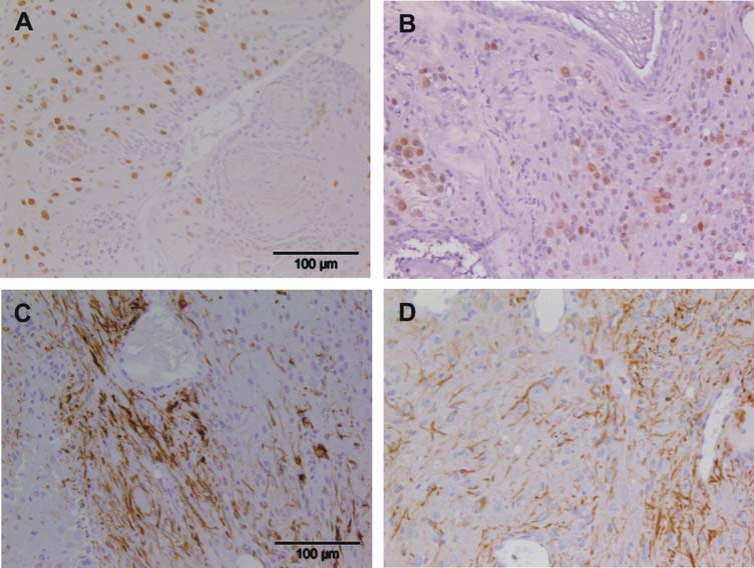
Immunohistochemical staining identifies neuronal and glial cells in teratomas derived from ES and *in vitro* differentiated cells. The tumors were grown in SCID/beige mice after injection of ES cells (A, C) or after injection of *in vitro* differentiated cells (B, D). The teratomas were stained by immunohistochemistry for the neuronal marker NeuN (A, B) and the glial marker GFAP (C, D) which is mainly found in astrocytes. In the sections shown here, groups of cells positive for NeuN and confluent groups of cells positive for GFAP were found.

To verify that the suppression of tumor growth in allogeneic hosts was indeed dependent on the immune system, the cells were injected into allogeneic SCID/beige mice which lack T and B lymphocytes as well as functional natural killer (NK) cells. In these recipients teratomas grew in similar frequency as in syngeneic hosts (Table 2). No significant difference of tumor growth was observed after injection of ES and differentiated cells (Fig. 1B). Interestingly, several teratomas of SCID/beige mice expressed OCT3/4 (Fig. 2G) in contrast to the tumors grown in syngeneic 129Sv mice (Table 3), suggesting a removal of those cells in immunocompetent mice. The teratomas grown after injection of ES cells and *in vitro* differentiated cells were stained for neuronal and glial markers (Fig. 4) and both cell types were found in a similar frequency (Table 4).

### PA6 feeder cells are not responsible for tumors observed after injection of differentiated cells

Differentiated cells were obtained by culturing MPI-II ES cells on a feeder layer of mitomycin-inactivated PA6 cells. We wanted to exclude that PA6 cells (derived from a C57BL/6 mouse) [13] could have contributed to the tumors. Therefore, we injected 1×10^6^ viable PA6 cells into groups of 6 to 8 C57BL/6, 129Sv, C3H/HeN, SCID/beige mice, and LOU/c rats. We did not observe any signs of tumor growth in these animals (data not shown). Thus, PA6 cells are not tumorigenic and do not contribute to the tumor growth after injection of differentiated cells.

### Effect of immunosuppression on tumorigenicity of ES cells and differentiated cells in various hosts

**Figure 5:**
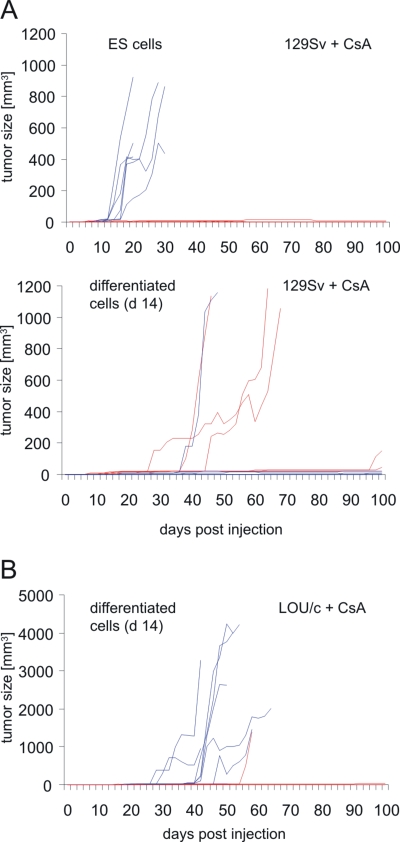
Tumor growth in CsA-treated 129Sv mice and LOU/c rats after injection of ES cells or *in vitro* differentiated cells. (A) 1×10^6^ ES cells or *in vitro* differentiated cells were injected subcutaneously at day 0 into syngeneic 129Sv mice (for the numbers of animals see Table 2) which were treated daily with CsA (10 mg/kg body weight). The tumor size was recorded every second day until day 100 using linear calipers. The growth of tumors in individual mice is shown. Tumor growth in female hosts is indicated by red lines and male hosts by blue lines. (B) 1×10^6^ cells differentiated *in vitro* for 14 days were injected subcutaneously into LOU/c rats (n = 18) which were treated daily with CsA (10 mg/kg body weight). The tumor size was recorded every second day until day 100 using linear calipers. The growth of tumors in individual rats is shown. Tumor growth in female hosts is indicated by red and in male hosts by blue lines. One tumor in a female rat was not progressive and remained very small until the end of the experiment. In two male rats a tumor regression occurred.

After experimental transplantation of ES cells immunosuppression is frequently used to avoid rejection of the transplanted cells. Therefore, we determined the effect of immunosuppression on the tumorigenicity of the cells. In 129Sv mice treated with CsA (10 mg/kg/day) we observed teratomas in a similar frequency as in untreated animals (Table 2). Interestingly, 8 of 10 female CsA-treated 129Sv mice injected with ES cells were able to reject the tumors within 100 days and in only one female a tumor was observed during autopsy. ES cells injected in parallel into male mice formed tumors in 6 of 6 cases (Fig. 5A). Thus, immunosuppression appeared to be associated with regression of ES cell-derived tumors in female 129Sv recipients. Differentiated cells formed tumors in 4 of 6 male and 6 of 6 female CsA-treated 129Sv mice. In CsA-treated mice tumor growth appeared to be accelerated in male animals after injection of ES cells (p<0.0180) and in female mice after injection of differentiated cells (p<0.0001). Thus, the tumor growth might depend on a complex interaction between the injected cell type and sex of the host (p<0.0001). We did not observe a similar interaction of these factors in the untreated 129Sv mice (p = 0.8198). The progressively growing teratomas expressed Ki67 but no OCT3/4 (Table 3). Interestingly, also the infiltration of the teratomas by T and B lymphocytes was not altered compared to the mice which did not receive CsA (Table 3).

In CsA-treated allogeneic C57BL/6 and C3H/HeN mice small tumors (<10 mm^3^) were detected in single cases (Table 2). However, injection of ES cells and differentiated cells did not lead to progressive tumor growth in CsA-treated allogeneic mice. Most surprisingly, injection of differentiated cells into CsA-treated LOU/c rats resulted in 11 of 18 cases in teratomas (Table 2). Tumor growth was observed in 9 of 9 male rats (Fig. 5B). In two of them an early tumor regression occurred. In addition, tumors were detected in 2 of 9 female rats within 100 days after inoculation (Fig. 5B). It is important to notice that none of the CsA-treated rats (including 12 males and 13 females) developed tumors after injection of ES cells (Table 2). Thus, the tumorigenicity of ES cells compared to differentiated cells was clearly reduced in CsA-treated rats (p<0.0001). After injection of differentiated cells the tumor growth was clearly dependent on the CsA treatment (p<0.0001) and on the sex of the host (p = 0.0127). The teratomas grown in the rats expressed Ki67 with exception of one non-progressive tumor. 5 of 9 teratomas expressed some OCT3/4 (Fig. 2H), as teratomas in immunodeficient mice did. However, it is not clear whether these OCT3/4-positive cells are ES cells from the grafts which survive when transplanted as contaminants of the tumorigenic differentiated cells or whether they represent another undefined cell population. All teratomas showed infiltration with rat T and B lymphocytes as well as rat macrophages (Table 3). Rat NK cells were rarely found in these fully established tumors (data not shown).

These experiments clearly demonstrate that tumorigenic cells are present among the *in vitro* differentiated cells which are not undifferentiated ES cells. Furthermore, it appears that the tumorigenicity of mouse ES cells in contrast to differentiated cells is controlled by a part of the rat's immune system which is not suppressed by CsA.

### Susceptibility of ES cells to natural cytotoxic cells and expression of ligands for inhibitory and activating NK receptors

**Figure 6:**
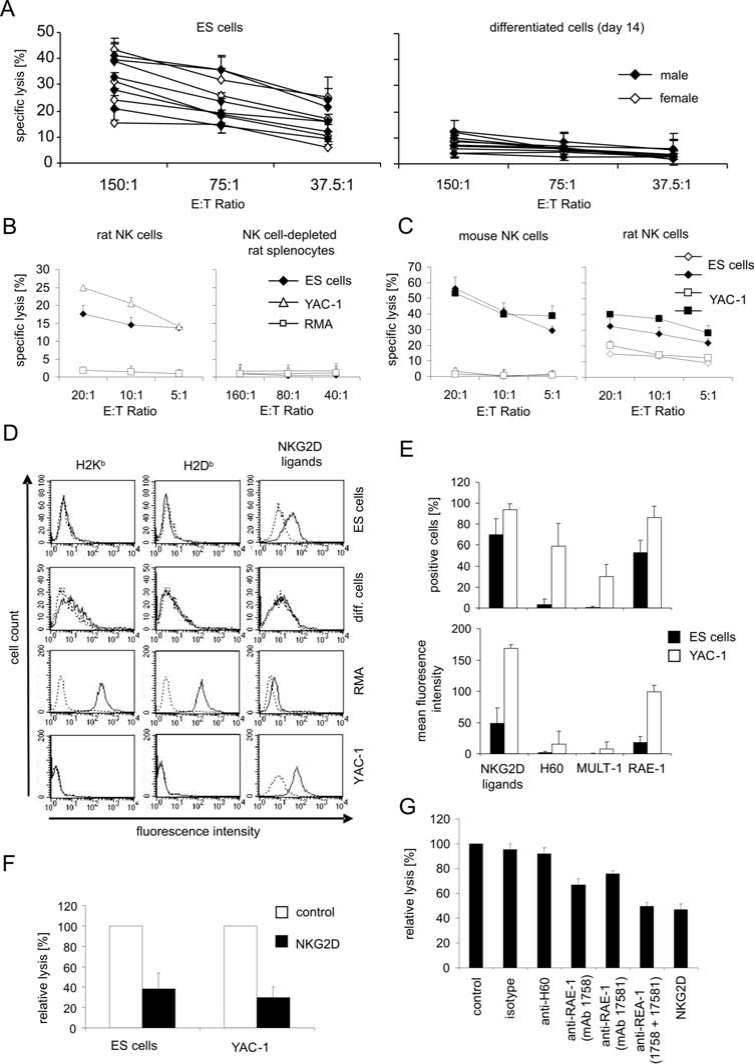
Lysis of ES and *in vitro* differentiated cells by NK cells derived from naïve LOU/c rats and expression analysis of MHC class I molecules and NKG2D ligands. (A) Mean of specific lysis and standard deviation (SD) of triplicates of ES or *in vitro* differentiated cells at three effector∶target (E∶T) ratios. Effector cells were lymphocytes obtained from spleens of 10 individual LOU/c rats by density gradient centrifugation on Biocoll. Results obtained with lymphocytes from female rats are indicated by open symbols and from male rats by filled symbols. (B) The mean specific lysis and SD of triplicates of ES, YAC-1, and RMA target cells at three effector∶target (E∶T) ratios by freshly isolated NK cells or NK cell-depleted splenocytes from naïve LOU/c rats are shown. The NK cell enriched fraction contained 86% and the NK cell depleted fraction 3% NKR-P1A-positive cells as determined by flow cytometry. The results are representative for 3 independent experiments. (C) The mean specific lysis and SD of triplicates of ES and YAC-1 cells at three effector∶target (E∶T) ratios by freshly isolated (open symbols) or 3 days *in vitro* with 1000 U/ml IL-2 stimulated (closed symbols) mouse and rat NK cells are shown. The NK cell-enriched fractions contained more than 80% NK cells as determined by flow cytometry. The results shown are representative for 3 independent experiments. (D) The expression of MHC class I molecules on ES and differentiated cells was analyzed by flow cytometry using anti-H2K^b^ and anti-H2D^b^ Abs (full lines). The stainings with the isotype control are shown by the dotted lines. RMA cells (H2^b^) served as positive control for these antibodies. NKG2D ligands were stained with a recombinant mouse NKG2D-Fc chimeric protein and a FITC-conjugated goat anti-human IgG antibody as secondary reagent (full lines). Stainings with the secondary reagent only are shown by the dotted lines. YAC-1 cells (H2^a^) served as positive controls for these stainings. The results shown are representative for more than 3 independent experiments. (E) ES and as positive control YAC-1 cells were stained with the NKG2D-Fc chimeric protein and with mAbs specific for the NKG2D ligands H60, MULT-1, and RAE-1. The mean+SD of the proportion of positive cells and the mean fluorescence intensity+SD determined in 6 independent experiments is shown. (F) The mean inhibition+SD of specific lysis of ES and YAC-1 cells by soluble mouse NKG2D is shown as determined in 3 experiments. The mean of specific lysis of the target cells by rat NK cells at an effector to target ratio of 20∶1 was determined as described above and adjusted to 100%. For inhibition of lysis a soluble mouse NKG2D protein was added to the test at a concentration of 3 µg/ml. The relative lysis of the target cells exposed to NKG2D was calculated. (G) The mean inhibition+SD of specific lysis of ES cells by soluble mouse NKG2D and various inhibitory antibodies against NKG2D ligands is shown. The mean of specific lysis of the target cells by IL-2 activated mouse NK cells at an effector to target ratio of 10∶1 was determined as described above and adjusted to 100%. For inhibition of lysis a soluble mouse NKG2D protein or the indicated mAbs were added to the test at a concentration of 3 µg/ml. The relative lysis of the target cells exposed to NKG2D or the mAbs was calculated.

**Figure 7:**
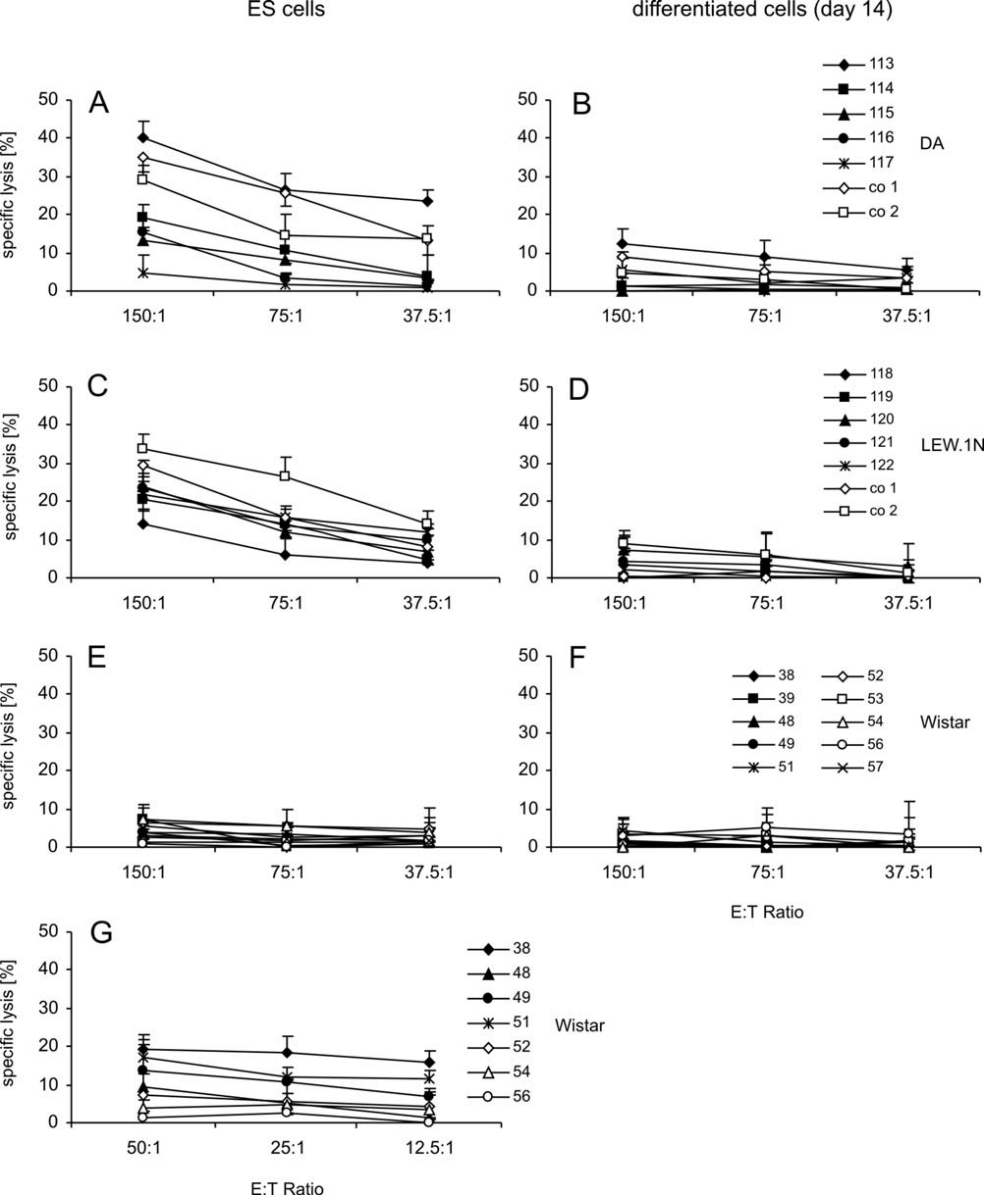
Lysis of ES and *in vitro* differentiated cells by splenocytes derived from rats 6 weeks after intracerebral grafting of *in vitro* differentiated cells. Mean of specific lysis and SD of triplicates of ES cells (A, C, E, G) or *in vitro* differentiated cells (B, D, F) at three effector∶target (E∶T) ratios. Effector cells were lymphocytes obtained by density gradient centrifugation on Biocoll from spleens of grafted (no. 113–117) or naïve (co1 and co2) DA rats (A, B), grafted (no. 118–122) or naïve (co1 and co2) LEW.1N rats (C, D), and grafted Wistar rats (no. 38, 39, 48, 49, 51, 52, 53, 54, 56, 57). The individual rats are indicated by symbols. (G) Mean of specific lysis and SD of triplicates of ES cells by lymphokine-activated killer (LAK) cells derived from splenocytes of Wistar rats (no. 38, 48, 49, 51, 52, 54, 56) after culture for 4 days in the presence of 100 U/ml IL-2.

CsA is known to suppress the adaptive immune system by interfering with T cell signaling. We suspected that a natural cytotoxic activity that is not suppressed by CsA was able to prevent the growth of tumors derived from ES cells but not from differentiated cells. Therefore, we analyzed the susceptibility of ES cells and differentiated cells to killing by splenocytes derived from naïve LOU/c rats. ES cells in contrast to differentiated cells were killed readily in chromium release assays by splenocytes from LOU/c rats (Fig. 6A). No differences in the cytotoxic activity between splenocytes of male and female rats were found. Lymphocytes derived from lymph nodes of the same animals did not show a cytotoxic activity against these targets (data not shown). Similar results were obtained when effector cells from other rat strains were used (see below Fig. 7). These results were in agreement with the assumption that NK cells, which are abundant in the spleen but rare in lymph nodes, can kill ES cells. We further isolated rat NK cells from spleens of naïve LOU/c rats by magnetic cell sorting of NKR-P1A-positive NK cells and used them directly without prior cytokine stimulation as effector cells in cytotoxicity assays against ES target cells. The purified NK cells were able to kill the ES cells and the typical NK target cell line YAC-1 in contrast to RMA cells which are known to be resistant to NK cells (Fig. 6B). The cytotoxic activity of the NK cells could be inhibited by ethyleneglycol-bis(b-aminoethyl ester)-N,N,N′,N′-tetraacetic acid (EGTA, data not shown), indicating that the granule exocytosis pathway was used to kill the targets. The NK cell-depleted splenocyte fraction was not able to kill any of these target cell lines even at much higher effector target ratios (Fig. 6B). Thus, the mouse ES cells were indeed susceptible to rat NK cells. We wondered why mouse NK cells were not able to suppress the teratoma growth in 129Sv mice after injection of ES cells. Interestingly, freshly isolated NK cells of naïve 129Sv mice completely failed to kill the ES cells. However, a short term culture in the presence of interleukin (IL)-2 was sufficient to activate them (Fig. 6C). Stimulation of rat NK cells with IL-2 increased also their preexisting cytotoxic activity against ES cells (Fig. 6C).

The cytotoxic activity of NK cells is known to be controlled by a set of inhibitory and activating NK receptors which are expressed on NK cells and interact with certain ligands on target cells. Ligands of inhibitory NK receptors are classical class I molecules, such as H2K and H2D. Ligands of activating NK receptors, such as NKG2D, include in the mouse the RAE-1 family as well as MULT-1 and H60 molecules. We determined the expression of classical MHC class I molecules (H2K^b^ and H2D^b^) and NKG2D ligands on ES and differentiated cells by flow cytometry (Fig. 6D). MHC class I molecules were not detectable on ES cells but slightly up-regulated during differentiation. NKG2D ligands showed clearly the opposite expression pattern and were expressed only in ES cells (Fig. 6D). The RAE-1 proteins were the NKG2D ligands expressed on the ES cells (Fig. 6E). NKG2D ligands on ES cells were most likely responsible for the susceptibility of these cells to rat NK cells since the NK cell-mediated killing could be blocked by a soluble NKG2D protein (Fig. 6F). Similarly, the killing by activated mouse NK cells could be inhibited by the soluble NKG2D protein and also by inhibitory antibodies against RAE-1 molecules (Fig. 6G).

### Cellular cytotoxicity against ES cells in rats after intracerebral grafting of differentiated cells

Rats are widely used as hosts to evaluate functional properties of cells which are differentiated *in vitro* from mouse ES cells [1], [2]. The differentiated cells analyzed here were evaluated previously for their therapeutic potential in a rat model of Parkinson's disease [3], [4]. In this model 10^5^ differentiated cells were transplanted into the striata of unilaterally 6-OHDA-lesioned rats. We now analyzed the cytotoxic activity of effector cells from transplanted rats 6 weeks after intrastriatal grafting. Rats from two inbred rat strains (DA and LEW.1N) and one outbred strain (Wistar) were used as recipients in these experiments. The animals did not receive any immunosuppressive therapy after transplantation. No teratomas were found in the brains of these animals (data not shown), confirming our previous observations [3].

Freshly isolated splenocytes from 4 of 5 grafted and two untreated control DA rats killed ES cells in chromium release assays at least to certain extend (Fig. 7A). A detectable killing is defined here as a mean specific lysis of more than 5% at least for the two highest effector target ratios. Differentiated cells were moderately killed only by cells from one grafted (no. 113) and one control (co 1) DA rat (Fig. 7B). Splenocytes obtained from all grafted and control LEW.1N rats killed ES cells (Fig. 7C). However, a low cytotoxic activity against differentiated cells was observed with splenocytes from only one grafted (no 120) and one control (co 2) LEW.1N rat (Fig. 7D). The cytotoxic activity of splenocytes from Wistar rats against ES cells appeared to be reduced compared to the other strains (Fig. 7E). Splenocytes from all animals failed to kill differentiated cells (Fig. 7F). However, a short term culture with IL-2 was usually sufficient to increase their cytotoxic activity against ES cells (Fig. 7G). Thus, a natural cytotoxic activity against mouse ES cells exists not only in LOU/c rats but also in other rat strains such as DA and LEW.1N.

### NK cells appear to contribute to the rejection of ES cells in vivo but further immune mechanisms might be involved

**Figure 8:**
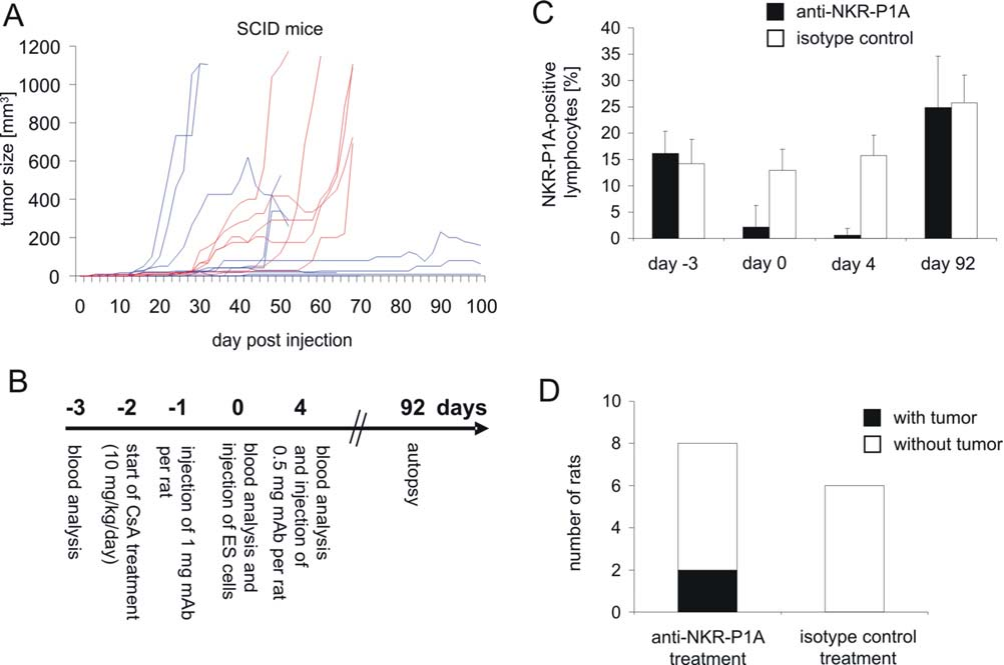
Analysis of effects of NK cells on the rejection of ES cells in vivo. (A) 1×10^6^ ES cells were injected subcutaneously at day 0 into 15 T and B cell deficient SCID mice which have functional NK cells. The tumor size was recorded every second day until day 100 using linear calipers. The growth of tumors in individual mice is shown. Tumor growth in female hosts is indicated by red lines and in male hosts by blue lines. In one animal a tumor regression was observed. (B) The treatment scheme of rats receiving an NK cells depleting antibody (anti-NKR-P1A) or an isotype control is shown. (C) The mean proportion plus SD of NK cells in the blood of male LOU rats receiving the anti-NKR-P1A mAb (n = 8) or the isotype control (n = 6) is shown. Blood cells were stained with the respective mAb and analyzed by flow cytometry after lysis of erythrocytes. (D) The proportion of the male rats in which tumors were found at autopsy is indicated. The size of the tumors was 12 mm^3^ and 16 mm^3^, respectively. Both were palpable for more than 30 days before autopsy.

The presence of NK cells with cytotoxic activity against ES cells in naïve and transplanted rats suggest that they might influence the outcome of the transplantation. However, in syngeneic 129Sv mice the ES cells gave rise to teratomas despite these mice have functional NK cells which can kill the ES cells *in vitro* after IL-2 stimulation. To determine whether allogeneic mouse NK cells can prevent teratoma growth *in vivo*, we injected the ES cells into SCID mice (C.B-17, H2^d^) which have no B and T cells but NK cells. In 100% (15/15) of the animals teratomas were observed within 100 days after injection of ES cells (Fig. 8A). Thus, the injection of ES cells alone might not be sufficient to activate the NK cells in mice. Therefore, we tried to inhibit the preexisting NK cell activity in rats. Unfortunately, in rats a genetic model of NK cell deficiency is not available. It has been shown that injection of high doses of monoclonal antibodies against the NKR-P1A molecule can transiently inhibit NK cell cytotoxicity *in vivo* [14]. We speculated that a transient inhibition of NK cells could be sufficient to allow for a differentiation of injected ES cells *in vivo* into a pluripotent and NK cell resistant cell population which could then give rise to teratomas. The treatment scheme and the efficiency of NK cell depletion are shown in Fig. 8B and 8C. In 2 of 8 male LOU rats small tumors were found at the injection site but none of the 6 control rats treated with an isotype control showed a tumor (Fig. 8D). Taking into account the limited efficiency of a transient NK cell inhibition, these data suggest that NK cells could contribute to the control of teratoma growth but likely further immune mechanisms are involved in the rejection of mouse ES cells in CsA-treated rats.

## Discussion

It is well known that mouse [8], [10], [15]–[18] and human ES cells [19], [20] can give rise to teratomas when injected into immunodeficient hosts. The ability of cells to form teratomas in such recipients can even be used as an *in vivo* test for their pluripotency [21]. Mouse ES cells can also form teratomas in syngeneic [9], [22], [23] and allogeneic hosts [8], [9], [17] and even in xenogeneic recipients such as rats [1], [5]. The frequency of tumors varied in these studies. This is probably depending on the ES cell lines used [24], the degree of histoincompatibility between transplanted cells and recipients, the immunosuppressive treatment of the recipients, and the site of transplantation [24], [25], especially when it is an immune-privileged organ. The situation is even more complex after transplantation of *in vitro* differentiated cells. Again teratomas have been observed in some studies in syngeneic [9], [22], allogeneic [6] and xenogeneic hosts [4], [5]. However, the results might have been also strongly influenced by the differentiation protocols and selection strategies [9]–[12].

We have used one ES cell line (MPI-II) and one differentiation protocol [3], [26] that after 14 days of culture results in about 95% Tuj1-positive neuronal cells and about 30% TH-positive dopaminergic cells [3], [26]. The same differentiation protocol has also been successfully used for the dopaminergic differentiation of human [27]–[29] and non-human primate ES cells [30], [31]. These differentiated mouse cells have been shown previously to improve the amphetamine-induced rotational behavior in unilaterally 6-OHDA-lesioned and not immunosuppressed rats after intrastriatal transplantation [3]. However, these cells gave rise to teratomas in 2 of 15 CsA-treated animals [4].

Subcutaneous injection of either 1×10^6^ ES cells or differentiated cells led to teratoma growth in about 95% of the syngeneic 129Sv recipients. It was surprising that neither frequency nor growth of the tumors derived from differentiated cells were significantly reduced. We have shown in a pilot experiment that a 10-fold reduction of the number of ES cells reduced the tumor frequency. The proportion of undifferentiated cells which still might have been present after *in vitro* differentiation was less than 10% [3]. Thus, it appears to be questionable whether only completely undifferentiated ES cells can contribute to the teratoma formation. In allogeneic mice and xenogeneic rats no tumor growth was observed. A mismatch in minor histocompatibility antigens was sufficient to suppress the tumorigenesis as shown in C57BL/6 recipients which share the MHC haplotype H2^b^ with 129Sv mice. Since both ES and differentiated cells formed teratomas in allogeneic immunodeficient SCID/beige mice the immune response was indeed responsible for the suppression of tumors after allogeneic transplantation.

Immunosuppression with CsA did not augment the tumorigenicity of the cells in allogeneic mice. However, this was different for xenogeneic rat recipients. ES cells were not able to form teratomas in CsA-treated rats but *in vitro* differentiated cells lead to progressive tumor growth in about 50% of the animals. This finding clearly indicates that differentiated cells must contain a tumorigeneic cell population that is different from the ES cells. These cells might be even more tumorigeneic than ES cells because this subpopulation of cells present in differentiation cultures gave rise to teratomas in a similar frequency as a homogenous ES cell population. The CsA-treatment which suppresses the activation of T lymphocytes does apparently not impair the rejection of ES cells. The rejection of the novel tumorigenic cell population among differentiated cells in non CsA-treated hosts must be mediated by a part of the immune system which is affected by CsA such as cytotoxic T lymphocytes (CTL). These observations challenge the common view that only completely undifferentiated ES cells can give rise to teratomas. Some cells which arise in the differentiation culture obviously retain pluripotency. The molecular characteristics of these cells are unknown. They might represent a physiological differentiation step or, alternatively, a pathological miss-differentiation might occur in some cells during the *in vitro* differentiation culture. We assume that a pluripotent cell population exists in the differentiation culture which is an intermediate between ES cells and neuronal cells. These cells must be different from neural rosette cells (R-NSC) because the histopathological evaluation of the tumors from CsA-treated rats indicated undoubtedly the growth of teratomas after injection of *in vitro* differentiated cells and not a “rosette overgrowth” that has been described recently after grafting of ES cell-derived R-NSCs [32].

Interestingly, the teratoma growth appeared to be accelerated in male immunocompetent syngeneic mice and xenogeneic rats. Therefore, the teratoma growth might be supported by male hormones. The risk of teratoma formation appears to be influenced by a complex interaction between sex and immune status of the recipients that needs further exploration. Therefore, it will be of interest to determine in further experiments the tumorigenicity of female ES cells in female as well as in male recipients.

Rather little is known about the immune response that occurs after transplantation of ES cells or cells differentiated from ES cells [33]–[35]. We speculated that NK cells might be involved in the rejection of ES cells in the CsA-treated rats, because their cytotoxic activity is not generally impaired by CsA, although in humans some effects of CsA on NK subpopulations have been described recently [36]. Furthermore, mouse as well as human ES cells might be a target for NK cells because they express no or low amounts of MHC class I molecules [8], [17], [37], [38] which serve as ligands for inhibitory NK receptors [39]. The expression of MHC class I molecules has been described to increase during *in vitro* differentiation of ES cells [40], [41]. Cells which express no MHC class I molecules might fail to inhibit the cytotoxic NK cell activity [42]. Human NK cells have been shown to kill human ES cell lines which expressed small amounts of ligands for the activating NK receptor NKp44 [37]. On the other hand it has been reported that mouse NK cells are not important for the rejection of human ES cells *in vivo* [19]. Alternatively, the complement system has been shown to contribute to the control of teratoma growth after transplantation of ES cells [18].

We found a high cytotoxic activity of splenocytes derived from naïve rats against the ES cells and a low activity against differentiated cells. Flow cytometry did not indicate the presence of MHC class I molecules (H2K and H2D) on these cells and the differentiation only moderately increased H2K expression. Interestingly, the ES cells expressed ligands for the activating NK receptor NKG2D. Recognition of these ligands by NKG2D on NK cells is known to trigger cytotoxicity [43] and the killing of the ES cells by rat NK cells as well as IL-2 activated mouse NK cells was indeed dependent on NKG2D. NKG2D ligands were down-regulated during differentiation culture and this finding explains the reduced susceptibility of these targets to natural cytotoxic cells much better than the observed moderate up-regulation of H2K molecules. These results could suggest that ES cells were rapidly destroyed in immunocompetent as well as in CsA-treated rat recipients by NK cells before teratomas were formed. Differentiated cells which are NK cell resistant but still tumorigenic could have given rise to teratomas in CsA-treated rats which fail to reject these cells due to the impaired adaptive immune system. Although an antibody-mediated NK cell depletion is not as effective as a genetic NK cell deficiency, our respective experiment resulted in small teratomas in a subgroup of rats, suggesting that NK cells might be involved in the rejection of ES cells in CsA-treated rats. However, in addition NK cells, likely further immune mechanisms contribute to the removal of ES cells. The unimpaired teratoma growth in SCID mice could even argue that mouse NK cells are not important at all for the rejection of ES cells [19]. However, one should notice that mouse NK cells had to be activated by IL-2 to become able to kill the ES cells *in vitro*. Therefore, the effect of mouse and rat NK cells on teratoma growth might depend on the degree of an inflammatory response after transplantation which can lead to NK cell activation.

NKG2D ligands appear to represent new markers for ES cells. Due to their expression at the cell surface they are potentially useful for sorting of ES and differentiated cells. NKG2D ligands include in the mouse RAE-1, H60, and MULT-1 [43]. These ligands are not expressed normally but they can become induced in response to stress, such as heat shock [44], virus infection [45], or genotoxic stress [46]. They might signal the presence of potentially dangerous cells to the immune system [47]. The expression of these ligands in tumor cells is known to contribute to the tumor surveillance by the immune system [43]. The NKG2D ligands found to be expressed on the ES cells belong mainly to the RAE-1 family. This finding is in accordance with one previous study, however, these authors reported that RAE-1 molecules on mouse ES cells do not confer susceptibility to lysis by NK cells [48]. It remains to be elucidated whether the expression of these ligands on ES cells has a physiological function. NKG2D ligands could allow, e. g., for the removal of displaced and potentially dangerous pluripotent cells by the immune system.

Recently it has been reported that mouse ES cells are resistant to antigen-specific CTL due to the expression of serpin-6 which is an endogenous inhibitor of granzyme B [40]. However, the MPI-II cells analyzed here were susceptible to granule exocytosis-mediated killing by rat NK cells and also by peptide-specific mouse CTL (own unpublished data). Thus, ES cells are not generally protected against cellular cytotoxicity. Human ES cell lines were also reported to be susceptible to human CTL [49]. It has to be investigated further whether ES cell lines vary in their susceptibility against the cytotoxic mechanisms of the immune system.

Much research efforts are currently directed towards the establishment of pluripotent cells from individuals which might be subsequently used for syngeneic transplantations before or after *in vitro* differentiation. These strategies might be associated with an increased risk of teratoma formation. Thus, it might be worthwhile to thoroughly evaluate whether allogeneic stem cells can be used as an effective and safe new therapeutic tool in regenerative medicine.

## Materials and Methods

### ES cell culture and neuronal differentiation culture

Undifferentiated mouse ES cells (MPI-II, 129Sv strain, XY karyotype) were expanded on gelatin-coated cell culture dishes in Glasgow minimal essential medium (GIBCO-Invitrogen, Karlsruhe, Germany) containing 1% fetal calf serum (FCS), 10% knockout replacement, 2 mM glutamine, 0.1 non essential amino acids, 1 mM sodium pyruvate, 0.1 mM 2-mercaptoethanol, 2000 U/ml leukemia inhibitory factor (GIBCO). The neuronal differentiation was induced in a culture on mitomycin C-inactivated PA6 feeder cells as described [3], [26]. On day 14 of the culture cells were used for experiments and are named throughout this study as “differentiated cells”.

### Culture of cell lines

The mouse lymphoma cell lines YAC-1 (H2^a^) and RMA (H2^b^) were maintained in NaHCO_3_-buffered Dulbecco's modified Eagle's medium (DMEM) supplemented with 10% FCS (Biochrom, Berlin, Germany), 2 mM L-glutamine, 1 mM sodium pyruvate, 100 U/ml penicillin, and 100 µg/ml streptomycin. PA6 feeder cells were cultured in the same medium on gelatin-coated cell culture plates.

### Animal experiments

Animals were bred in the central animal facility of the Medical Faculty of the University of Göttingen. Animal experiments had been approved by the local government. Rats (DA, LEW.1N, LOU/c, Wistar) and immunocompetent mice (129Sv, C3H/HeN, C57BL/6) were conventionally housed whereas severe combined immunodeficient SCID/beige (C.B-17/IcrHsd-scid-bg) and SCID (C.B-17/Ztm-scid) mice were kept under pathogen-free conditions. A subgroup of animals received daily intraperitoneal injections of CsA (10 mg/kg, Sandimmune, Novatis Pharma, Nürnberg) starting two days before grafting. For the depletion of NK cells, some rats received in addition to CsA intraperitoneal injections of the monoclonal antibody (mAb) anti-NKR-P1A (clone 10/78, mouse IgG_1_, BD Biosciences, Heidelberg, Germany) or the respective isotype control (clone PPV-06, mouse IgG_1_, Exbio, Prague, Czech Republic). The anti-NKR-P1A mAb (clone 10/78) is directed against the same epitope as the mAb (clone 3.2.3) which has been used previously to deplete NK cells in rats [14]. One mg of the respective antibodies were given one day before the injection of ES cells followed by 0.5 mg at day 4 after cell transplantation. Blood samples were taken before starting these experiments, at day 0 and 4 days after cell transplantation, and at autopsy (day 92) in order to determine the proportion of NK cells in the blood by flow cytometry. For the analysis of subcutaneous tumor growth ES or differentiated cells were injected in 100 µl phosphate-buffered saline (PBS) into the flank of the animals. Tumor growth was monitored every second day by palpation and size was recorded using linear calipers. The tumor volume was calculated by the formula V = πabc/2, where a, b, c are the orthogonal diameters. Animals were sacrificed before day 100 when a tumor volume of 1 cm^3^ in mice and 5 cm^3^ in rats was reached, when a weight loss of more than 10% occurred, or when any behavioral signs of pain or suffering were observable. Autopsies of all animals were performed. Tumor tissue was immediately frozen in liquid nitrogen or placed in phosphate-buffered 4% formalin for 16 h and then embedded in paraffin. Spleens and lymph nodes were removed for subsequent immunological analyses. The transplantation of cells into the striatum of unilaterally 6-OHDA-lesioned rats was performed as previously described [3]. These animals were sacrificed 6 weeks after transplantation.

### Histology, immunohistochemistry, and immunofluorescence

Tissue sections (2.5 µm) were stained with hematoxylin and eosin (HE) for histological examinations. For immunohistochemistry, intrinsic peroxidase activity was blocked by incubation with 5% H_2_O_2_ in PBS for 20 minutes after deparaffinization. Non-specific antibody binding was inhibited with 10% FCS in PBS for 25 minutes. Microwave pre-treatment for antigen retrieval was regularly performed with exception of the sections for the NeuN and glial fibrillary acidic protein (GFAP) stainings. Immunohistochemical staining was performed with an avidin-biotin technique. The primary mAbs for immunohistochemistry were anti-NeuN (1∶100, clone MAB277, mouse IgG_1_, Chemicon, Temecula, CA, USA), anti-GFAP (1∶50, clone 6F2, mouse IgG_1_, Dako, Hamburg, Germany), anti-mouse CD3 (1∶200, clone CD3-12, rat IgG_1_, Serotec, Düsseldorf, Germany), anti-rat CD3 (1∶500, clone 1F4, mouse IgM, Serotec), anti-mouse CD45R/B220 (1∶200, clone RA3-6B2, rat IgG_2a_, BD Biosciences,), anti-rat CD45R (1∶200, clone HIS24, mouse IgG_2b_, BD Biosciences), anti-mouse Mac3 (1∶200, clone M3/84, rat IgG_1_, BD Biosciences), anti-rat CD68 (1∶500, clone ED1, mouse IgG_1_, Serotec), anti-Ki67 (1∶20, clone Tec3, rat IgG_2a_, Dako), and anti-OCT3/4 (1∶50, clone 40, mouse IgG_1_, BD Biosciences). Rat natural killer cells were stained on frozen sections (5 µm) using an anti-NKR-P1A mAb (clone 10/78, mouse IgG_1_, BD Biosciences). Immunofluorescence stainings of colonies of *in vitro* differentiated cells were performed and quantified as described before [3] using mAbs against OCT3/4, Ki67, and Tuj1 (1∶200, clone TUJ1 1-15-79, rabbit IgG, CRP Inc., Berkeley, CA, USA). To roughly estimate the frequency of OCT3/4-positive colonies after differentiation, 200 randomly chosen colonies were screened and the stained colonies were counted.

### Flow cytometry

Flow cytometry was performed on a FACScan™ flow cytometer (BD Biosciences) using CellQuest™ software. The cell surface expression of MHC class I molecules was analyzed using locus and haplotype-specific mAbs anti-H2K^b^ (clone CTKb, mouse IgG_2a_, phycoerythrin (PE)-conjugated, Caltag Laboratories, Hamburg, Germany), and anti-H2D^b^ (clone CTDb, mouse IgG_2a_, PE-conjugated, Caltag). A recombinant mouse NKG2D-Fc chimeric protein (139-NK; R&D Systems) was used to detect cell surface expression of NKG2D ligands. A polyclonal fluorescein isothiocyanate (FITC)-conjugated goat anti-human IgG (109-095-098; Jackson Laboratories, Dianova, Hamburg, Germany) served as secondary reagent. The following mAbs were used to detect specific NKG2D ligands: anti-RAE-1 (pan-specific, clone 186107, rat IgG2a, R&D Systems), anti-H60 (clone 205326, rat IgG2a, R&D Systems), and anti-MULT-1 (clone 237104, rat IgG2a, R&D Systems). A polyclonal FITC-conjugated goat anti-rat IgG (212-095-082; Jackson Laboratories, Dianova) served as secondary reagent. The purity of rat NK cells separated by magnetic cell sorting (MACS) and the proportion of NK cells in the blood of rats were assessed using an anti-NKR-P1A mAb (clone 10/78, mouse IgG1, PE-conjugated, Caltag). Erythrocytes in blood samples were lysed before analysis using a FACS lysing solution (BD Biosciences) according to the manufactures instructions. The purity of MACS-separated mouse NK cells was assessed using the pan-NK cell marker CD49b (clone DX5, rat IgM, PE-conjugated, Caltag). Isotype controls were purchased from Caltag.

### Effector cells and ^51^Chromium release assays

Lymphocytes were obtained from spleens or lymph nodes using a Tenbroeck homogenizer. Erythrocytes were removed from splenocytes by density gradient centrifugation on Biocoll (Biochrom). The cells were used either directly as cytotoxic effector cells or cultured for 4 days in DMEM supplemented with 10% FCS, 50 µM 2-mercaptoethanol, and 1000 U/ml IL-2 (Chiron, Amsterdam, Netherlands) to obtain lymphokine-activated killer (LAK) cells. Rat NK cells were isolated by MACS using a biotin-conjugated anti-NKR-P1A Ab (clone 10/78, mouse IgG_1_, BD Biosciences) and paramagnetic anti-biotin micro beads (130-090-485, Miltenyi Biotec, Bergisch-Gladbach, Germany) according to the manufactures instructions. Mouse NK cells were isolated using a negative selection kit (NK cell isolation Kit mouse, 130-090-864; Miltenyi Biotec). The kit contains a cocktail of antibodies against CD4, CD5, CD8a, CD19, Ly-6G, and Ter-119 and depletes the non-NK cells. To activate NK cells *in vitro* they were cultured in DMEM supplemented with 10% FCS, 50 µM 2-mercaptoethanol, and 1000 U/ml IL-2. ^51^Chromium release assays were performed as previously described [50], [51]. A recombinant mouse NKG2D-Fc chimeric protein (139-NK) purchased from R&D Systems (Wiesbaden, Germany) was used to inhibit the part of lysis of target cells which was dependent on the expression of NKG2D ligands. Similarly, antibodies which block the binding of NKG2D to RAE-1β (MAB17581, clone 199205, rat IgG_2a_, R&D Systems), RAE-1α and RAE-1γ (MAB 1758, clone 199215, ratIgG_2a_, R&D Systems), and H60 (clone 205326, rat IgG_2a_, R&D Systems) were used for blocking experiments. A rat IgG_2a_ isotype control was purchased from Caltag.

### Statistics

A nonparametric ANOVA-like method for longitudinal data based on ranks was used [52] to compare the tumor growth in different experimental groups. Due to numerical issues we compared tumor growth curves longitudinally using the following time points: 10, 20, 30, 40, and 50 days post injection.
